# Identification of Candidate Genes for Calcium and Magnesium Accumulation in *Brassica napus* L. by Association Genetics

**DOI:** 10.3389/fpls.2017.01968

**Published:** 2017-11-15

**Authors:** Thomas D. Alcock, Lenka Havlickova, Zhesi He, Ian Bancroft, Philip J. White, Martin R. Broadley, Neil S. Graham

**Affiliations:** ^1^Plant and Crop Sciences Division, University of Nottingham, Loughborough, United Kingdom; ^2^Department of Biology, University of York, York, United Kingdom; ^3^The James Hutton Institute, Dundee, United Kingdom; ^4^Distinguished Scientist Fellowship Program, King Saud University, Riyadh, Saudi Arabia

**Keywords:** associative transcriptomics, GWAS, *Brassica napus*, calcium, magnesium, biofortification, nutrient use efficiency

## Abstract

Calcium (Ca) and magnesium (Mg) are essential plant nutrients and vital for human and animal nutrition. Biofortification of crops has previously been suggested to alleviate widespread human Ca and Mg deficiencies. In this study, new candidate genes influencing the leaf accumulation of Ca and Mg were identified in young *Brassica napus* plants using associative transcriptomics of ionomics datasets. A total of 247 and 166 SNP markers were associated with leaf Ca and Mg concentration, respectively, after false discovery rate correction and removal of SNPs with low second allele frequency. Gene expression markers at similar positions were also associated with leaf Ca and Mg concentration, including loci on chromosomes A10 and C2, within which lie previously identified transporter genes *ACA8* and *MGT7*. Further candidate genes were selected from seven loci and the mineral composition of whole *Arabidopsis thaliana* shoots were characterized from lines mutated in orthologous genes. Four and two mutant lines had reduced shoot Ca and Mg concentration, respectively, compared to wild type plants. Three of these mutations were found to have tissue specific effects; notably reduced silique Ca in all three such mutant lines. This knowledge could be applied in targeted breeding, with the possibility of increasing Ca and Mg in plant tissue for improving human and livestock nutrition.

## Introduction

Calcium (Ca) and magnesium (Mg) are essential plant nutrients and vital for human and animal nutrition (Broadley and White, 2010; White and Brown, 2010). In plants, most Ca is extracellular, where it is a key strengthening component in cell walls (Grusak et al., 2016). It also has an important role in plant-cell signaling. Calcium enters root cells through a variety of Ca^2+^-permeable cation channels (White and Broadley, 2003; Karley and White, 2009; White, 2015). The opening of these channels must be tightly controlled, as changes in cytosolic Ca^2+^ concentrations coordinate numerous developmental and environmental stress responses (McAinsh and Pittman, 2009). The accumulation of Ca at tissue- and cellular-levels is dependent on the expression of transport proteins (Conn and Gilliham, 2010; Rios et al., 2012). After uptake from soil by roots, Ca travels via either apoplastic or symplastic pathways to the xylem, through which, in the form of either Ca^2+^ or complexed with organic acids, it is transported to the shoot. Calcium is immobile in the phloem, and as such, tissues with low transpiration rates (including fruits, seeds, and tubers) often have low Ca concentrations (Karley and White, 2009). Among plant nutrients, Ca is required in relatively large amounts. However, concentrations vary amongst taxa, typically ranging from ∼0.1 to 4.4% dry matter (Broadley et al., 2003). Calcium deficiencies are relatively rare in field-grown crops, but can occur in crops grown in acidic or leaching prone soils. Where Ca supply is insufficient to meet growth requirements, costly symptoms can ensue. For instance, fruits lacking in Ca are prone to cracking, as a direct result of weakness in the cell wall (White, 2015).

Magnesium is essential for photosynthesis, forming the central atom of chlorophyll molecules. It also has a key role in protein synthesis by functioning as a bridging element for the aggregation of ribosome subunits, as well as in photophosphorylation and generation of reactive oxygen species in plants (Cakmak and Yazici, 2010). Magnesium is taken up by roots as Mg^2+^. Control of influx across the plasma membrane is dominated by members of the MGT/MRS2 family of transport proteins and potentially Mg^2+^-permeable cation channels (Karley and White, 2009; Lenz et al., 2013). One member of the MGT gene family in *Arabidopsis thaliana*, *MAGNESIUM TRANSPORTER 1* (*MGT1*), encodes a protein localized to the plasma membrane (Li et al., 2001), suggesting its importance in the import and/or export of Mg in cells. Like Ca, Mg is transported from root to shoot cells through the xylem either as Mg^2+^ or complexed with organic acids. However, Mg is a phloem-mobile element, and is readily translocated to fruit, seeds, and tubers (White and Broadley, 2009). Shoot Mg concentrations are typically lower than shoot Ca concentrations across plant taxa, and vary between ∼0.1% to ∼1.0% dry matter (White et al., 2015).

In humans and animals, Ca is associated with the formation and metabolism of bone as well as being crucial for mediating vascular contraction and vasodilation, muscle function, nerve transmission, intracellular signaling, and hormonal secretion (Catharine, 2011). Based on food supply data, it was estimated that half of the population worldwide was at risk of Ca deficiency in 2011, with significant deficiency risks across all continents (Kumssa et al., 2015b). Magnesium is needed for over 300 biochemical reactions. It helps to maintain muscle function, prevents an irregular heartbeat, and is involved in protein synthesis (Yardley, 2009). Based on food supply data, <1% of the global population appeared to be at risk of dietary Mg deficiency in 2011 (Kumssa et al., 2015a). However, these data do not account for inhibitors of Mg adsorption, household waste, or distribution within countries and it is likely that significant deficiency risks exist within some populations. Magnesium deficiency risks are also likely to be greater in higher-income groups consuming processed foods, because Mg is among the nutrients commonly lost in processing (Swaminathan, 2003; Broadley and White, 2010; Kumssa et al., 2015a). Biofortification of crops has been previously suggested as a suitable approach for alleviating human deficiencies in a number of mineral nutrients, including Ca and Mg (White and Broadley, 2005; White and Broadley, 2009).

Previous analyses of variation in mineral concentrations across a wide range of plant species have shown that tissue Ca and Mg concentrations are inherently high in Brassicaceae compared most other taxa (Broadley et al., 2004; White et al., 2015). These traits have proven to be heritable in *Brassica oleracea* (Broadley et al., 2008), *B. rapa* (Graham et al., 2014), and *B. napus* (Thomas et al., 2016). Thus, *Brassica* spp. are potentially good targets for understanding genetic bases of leaf Ca and Mg accumulation, and for potentially increasing dietary intakes of Ca and Mg in humans and animals. Expression quantitative trait locus (eQTL) analyses in *B. rapa* previously led to the discovery of Ca responsive genes which may prove useful in marker-assisted selection for increased Ca concentration in shoot tissue (Graham et al., 2014). These include orthologs of *A. thaliana* Ca^2+^ transporter genes *CATION EXCHANGER 1* (*CAX1*) and *AUTOINHIBITED CA^2+^ ATPASE, ISOFORM 8* (*ACA8*), and subsequent work showed that allelic variants of the former gene in *B. rapa* influenced Ca accumulation. *B. napus* includes oilseed types, swedes and fodder crops, and is widely cultivated globally. It is an amphidiploid species that likely originated from multiple spontaneous hybridizations between *B. rapa* (A genome; turnip rape) and *B. oleracea* (C genome; cabbage, kale) and contains a full set of chromosomes from each (Iniguez-Luy and Federico, 2011; Chalhoub et al., 2014). This complexity has previously hindered the genetic study of this and other polyploid crops. However, recent and ongoing advances in sequencing and genome mapping technologies have allowed the rapid genotyping of multiple accessions at a fraction of the cost of older technologies. This has improved the feasibility of using a large diversity population over traditional mapping populations in genetic studies of crop species (Trick et al., 2009).

Associative transcriptomics (Harper et al., 2012) focuses on the analysis of transcribed sequences (mRNA-seq) across diversity populations to identify high-resolution loci influencing complex traits. An advantage using of RNA over DNA sequences in association studies is the ability to develop markers based on both single-nucleotide polymorphisms (SNPs) and transcript abundance (gene-expression markers; GEMs; Harper et al., 2012). Gene expression levels may be particularly important in the control of traits in polyploid species in which gene duplication may have led to unequal expression (Adams et al., 2003). Associative transcriptomics has been recently used in *B. napus* to identify genes underlying control of seed glucosinolate content (Harper et al., 2012; Lu et al., 2014) and anion homeostasis (Koprivova et al., 2014). The former two studies utilized panels of 84 and 101 genotypes, respectively. Despite the relatively small population sizes, a number of loci associated with seed glucosinolate concentrations were identified. Most notable associations include loci containing orthologs of *A. thaliana HIGH ALIPHATIC GLUCOSINOLATE 1* and *3* (*HAG1* and *HAG3*), known to regulate aliphatic glucosinolate biosynthesis (Sønderby et al., 2010). Koprivova et al. (2014), also made use of the panel of 84 genotypes and identified a number of loci associated with leaf nitrate, phosphate, and sulfate. Within these loci were a number of clear candidate genes, including a calcium-activated chloride channel previously shown to control nitrate levels in *A. thaliana* (De Angeli et al., 2006) which was associated with leaf nitrate concentration and a hypothetical phosphate/phosphoenolpyruvate translocator associated with leaf phosphate concentration.

Leaf Ca and Mg concentrations were previously characterized in a diversity population of ∼400 genotypes of *B. napus* in a broad-spectrum mineral analysis (Thomas et al., 2016). This population is likely to capture most of the species-wide variation, comprising oilseed, swede and fodder types. In this study, we perform associative analyses on this data using transcriptome sequences from 383 genotypes to identify genes influencing Ca and Mg accumulation. Candidate genes could be applied in marker assisted breeding in this and other *Brassica* crops, with the possibility of improving nutrient use efficiency of the crop and increasing available nutrients in edible plant tissue for improving human and livestock nutrition.

## Materials and Methods

### Characterization of Leaf Ca and Mg Concentration

This study used the Renewable Industrial Products from Rapeseed (RIPR) diversity population of inbred lines of *Brassica napus* genotypes (Thomas et al., 2016). These were developed from the ERANET-ASSYST consortium diversity population (Bus et al., 2011, 2014; Körber et al., 2012, 2015) with further lines included. A subset of 383 genotypes were selected, comprising 160 winter-, 127 spring-, and seven semi-winter-oilseed rape (OSR), 35 swede, 15 winter fodder, and 39 exotic/unspecified habits. These were previously characterized for leaf mineral concentrations by inductively coupled plasma-mass spectrometry (ICP-MS) of polytunnel-grown plants sampled at the rosette stage (typically 6–8 true leaves showing; Thomas et al., 2016). The full leaf mineral dataset is available at the Brassica Information Portal (BIP^1^; The Earlham Institute, Norwich, United Kingdom) and at doi: 10.5281/zenodo.59937.

### Associative Analyses

#### Transcriptome Sequencing and Population Structure Analysis

Extraction of RNA, quality checking and Illumina transcriptome sequencing were carried out as described by He et al. (2016). Tissue samples for RNA extraction were prepared from second true leaves, harvested when they reached ∼3 cm in diameter. RNA-seq data from each accession was mapped using methods described by Bancroft et al. (2011) and Higgins et al. (2012) onto ordered *Brassica* A and C genome-based pan-transcriptomes developed by He et al. (2015). Transcriptome sequencing was performed by the Earlham Institute (formerly The Genome Analysis Centre; Norwich, United Kingdom). Across the 383 accession panel, 46,307 single SNPs and 309,229 hemi-SNPs were detected and scored of which 256,397 SNPs had a population second allele frequency (saf) > 0.01. Transcript abundance was quantified and normalized as reads per kb per million aligned reads (RPKM) for each accession for 116,098 coding DNA sequence (CDS) models of the pan-transcriptome reference. Significant expression (mean > 0.4 RPKM) was detected for 53,889 CDS models. Inference of population structure by Q-matrix was obtained by Population Structure Inference using Kernel-PCA and Optimization (PSIKO; Popescu et al., 2014). A heatmap illustrating the relatedness of all genotypes in this study can be found in Supplementary Figure 1. Transcriptome sequences are deposited within the Sequence Read Archive (Leinonen et al., 2011) under accession number PRJNA309367.

#### Associative Transcriptomics

Associative transcriptomics was performed using SNPs, Q-matrix and trait data in a compressed mixed linear model approach (Zhang et al., 2010) implemented in the GAPIT R package (Lipka et al., 2012) in R 3.2.0 (R Core Team, 2015). The association analysis between gene expression markers (GEMs) and traits was performed by using fixed effect linear modeling in R with RPKM values and Q-matrix data as the explanatory variables and trait score the response variable using custom designed scripts (Harper et al., 2012; Havlickova et al., in press). Coefficients of determination (*R*^2^), constants and significance values were calculated for each regression. Manhattan plots were generated using graph functions in R. SNPs with low second allele frequency (<0.01) were filtered from the dataset prior to generating plots. In total 256,397 SNPs and 53,889 GEMs were plotted. False discovery rate (FDR; Benjamini and Hochberg, 1995) and Bonferroni (Dunn, 1961) corrections were used to set significance thresholds at *P* < 0.05. Due to sequence similarity between *B. napus* A and C genomes, assignment to a specific genome was not possible for all SNP markers; such markers are plotted in gray and appear in both positions on Manhattan plots. See Supplementary Figures 2–5 for quartile–quartile (QQ) plots of each association analysis.

#### Candidate Gene Identification

Ordered pan-transcriptome data based on *Brassica* A and C genomes from *B. rapa*, *B. napus*, and *B. oleracea* CDS gene models (He et al., 2015) were used to identify candidate genes. Candidate genes were selected based on *Arabidopsis thaliana* annotated functions of *Brassica* orthologs within estimated linkage disequilibrium (LD) decay of significantly associated markers (around 1–2 cM on average; Ecke et al., 2010). Further information relating to candidate gene predicted function was obtained from genome browsers comprising sequences of *B. rapa* (A genome, Chiifu-401-41; Wang et al., 2011) and *B. oleracea* (C genome, TO1000DH3; Parkin et al., 2014) at Ensembl Plants (Kersey et al., 2016). *A. thaliana* functional information were obtained from The Arabidopsis Information Resource (TAIR; Huala et al., 2001). Further resources used to aid with selection of candidates included *A. thaliana* gene expression data at The Bio-Analytic Resource for Plant Biology (Waese and Provart, 2017) and ionomic data at the Purdue Ionomics Information Management System (PIIMS; Baxter et al., 2007).

### Experiments Using *Arabidopsis thaliana* Mutants

#### Plant Material and Genotyping

Seed of 15 *Arabidopsis thaliana* mutant lines representing 10 candidate genes were acquired from the Nottingham Arabidopsis Stock Centre (Nottingham, United Kingdom). These comprised SALK (Alonso et al., 2003) and SAIL (Sessions et al., 2002) T-DNA lines and are summarized in **Table 1**. *Arabidopsis thaliana* ecotype Columbia-0 (Col-0) was used as the wild type control in all experiments. Plants were by genotyped for homozygous T-DNA insertions by conventional PCR. Genotyping primers are summarized in **Table 1**. Left border primers used were SALK LBb1 and SAIL LB1 for SALK and SAIL lines, respectively.

**Table 1:** Summary of *Arabidopsis thaliana* T-DNA insertion lines acquired for characterisation including primers used for genotyping.

Line Name	SALK/SAIL code	NASC stock code	Forward primer	Reverse primer
At2g05120.1	SALK_119762	N619762	TTCTGGAGAAACAAGGTCCAA	ATGGCAGCAAGTTTTTCACC
At2g13610.1	SALK_074250	N574250	CGATTTGCCGAAAAGAAAAA	GTTTCCTCCACCGTAAGCAA
At2g13610.2	SALK_074250C	N681303	CGATTTGCCGAAAAGAAAAA	GTTTCCTCCACCGTAAGCAA
At2g45660.1	SALK_138131	N638131	GGTTCTTCCTTTCGCAGAGA	CCACAAAAGGCCAATCAAAT
At5g03960.1	SALK_138382	N638382	TGGTTGAGGAAGCAAGAAGG	TGTGCTCTGCCTCCTTTGTA
At5g06530.1	SALK_024391	N524391	TTCCCCAAAGGTATCGATTCTA	TCGAACAACTGGGATTGACA
At5g06530.2	SALK_076250	N576250	TTCCCCAAAGGTATCGATTCTA	CGGGCATTTGATAGCACTTT
At5g07320.1	SALK_037517	N537517	CGCTGCATATGAAACGCTAA	TCAATGATCGCAACAAAACAA
At5g07320.2	SALK_037517C	N683966	CGCTGCATATGAAACGCTAA	CCATAAAAATATATGTCCCAATTTCA
At5g08670.1	SALK_083107	N583107	CGATGTTCCCAACATTTGAA	AACAGAGACCGGCGAGACTA
At5g10520.1	SALK_019299	N519299	TATTTCATGCACGGCATTGT	GGGTTGGAAATGTGGAAGAA
At5g10520.2	SALK_053754	N553754	CCGTTTCGTCTTCTCACCAT	ACATGGTGAGGCCAGTTCTC
At5g14040.1	SALK_105845	N605845	CCCTTACTTTTCGGAGCATTC	TTGCACTTGACGAGATCGAG
At5g48650.1	SALK_027468	N527468	GCGGTAGCTGAGGGTACATC	CCACCATCAAGCCAAAGACT
At5g48650.2	SAIL_64_G12	N803057	GCCCAATAGGCAAACAAATG	AAGTCTGGGACCAACAATGG

*NASC stock code shown for reordering*.

#### Preliminary Phenotyping

Seeds from homozygous mutant lines and Col-0 were sterilized in bleach, then washed in H_2_O and 70% ethanol prior to sowing on plates containing 1% agar containing 0.4 g L^-1^ Hoagland’s solution (Hoagland and Arnon, 1950; ¼ strength). Plates were stored in darkness at 4°C for 24 h, and then moved to a controlled environment growth chamber set to 23°C (∼30 W m^-2^ continuous light). After 7 days, plants were transferred to pots containing Levington M3 compost (ICL Specialty Fertilizers, Ipswich, Suffolk, United Kingdom) plus T34 biocontrol (Fargro Ltd., Arundel, West Sussex, United Kingdom) and placed on flow benches in a glasshouse with 18°C heating, venting at 20°C, with 16-h supplementary lighting (76 W m^-2^). Flow bench automatic irrigation operated once daily. After 10 days of establishment, six plants of each line were chosen randomly and transferred to individual wells in 16 well trays in a six block, using a one-way randomized design generated in GenStat (17th edition; VSN International, 2014) in which plants of each line were represented once per block and randomized within each block (Supplementary Table 1). In total, each line was represented six times. ARACON systems (Betatech BVBA, Gent, Belgium) were used to keep plants separate. At mid-flowering, whole shoots were harvested by cutting them below the rosette. Shoots were dried at 50°C for at least 2 days, and then crushed by hand within paper bags. Shoot subsamples (∼0.10 g DW) were digested using a microwave system comprising a Multiwave 3000 platform with a 48-vessel MF50 rotor (Anton Paar GmbH, Graz, Austria). Digestion vessels were perfluoroalkoxy (PFA) liner material and polyethylethylketone (PEEK) pressure jackets (Anton Paar GmbH). Leaf material was digested in 2 mL 70% Trace Analysis Grade HNO_3_, 1 mL Milli-Q water (18.2 MΩ cm; Fisher Scientific UK Ltd., Loughborough, United Kingdom), and 1 mL H_2_O_2_ with microwave settings as follows: power = 1,400 W, temp = 140°C, pressure = 2 MPa, time = 45 min. Two operational blanks and duplicate samples of certified reference material (CRM; Tomato SRM 1573a, NIST, Gaithersburg, MD, United States) were included in each digestion run. Following digestion, each tube was made up to a final volume of 15 mL by adding 11 mL Milli-Q water and transferred to a 25 mL universal tube (Sarstedt Ltd., Nümbrecht, Germany) and stored at room temperature. Leaf digestates were diluted 1-in-5 using Milli-Q water prior to broad-spectrum elemental analysis by ICP-MS as described previously (Thomas et al., 2016). For each data-point, an element-specific operational blank concentration (mean of each ICP-MS run) was subtracted. Data were then multiplied by initial sample volume, divided by the initial dry mass of plant material, and converted to mg element kg^-1^ of dry leaf or seed material. The CRM Ca and Mg recovery averaged 99 and 89%, respectively.

#### Tissue Partitioning Experiment

Based on results from preliminary phenotyping, lines At2g13610.2, At5g07320.2, and At5g48650.2 were found to have significantly lower shoot Ca or Mg concentrations than wild type plants and hence were selected for further characterization. Individual seed from these lines and Col-0 were sown into 12 well trays containing Levington M3 compost plus T34 biocontrol and placed on flow benches in a glasshouse with 18°C heating, venting at 20°C, with 16-h supplementary lighting (76 W m^-2^). Flow bench automatic-irrigation operated once daily. After successful establishment, 12 plants per genotype were selected randomly and transplanted into individual 9 cm pots. These were arranged in a 12 block, one-way randomized design generated in GenStat in which each genotype was represented once per block and genotypes randomized within each block (Supplementary Table 2). At mid-flowering (40 days after sowing), entire shoots were harvested. Shoots were partitioned into rosette leaves, stem, cauline leaves, and siliques. Tissue samples were dried at 50°C for 6 days, and then samples from plants in blocks 1–4, 5–8, and 9–12 were pooled into the four genotypes to ensure enough sample was available for mineral analysis. Pooled samples were crushed by hand, and then microwave digested prior to mineral analysis by ICP-MS as described above. Digestates were diluted 1-in-10 prior to mineral analysis. The recovery of Ca and Mg from the CRM averaged 96 and 88%, respectively.

#### Statistical Analyses

Data from experiments using *A. thaliana* mutants were analyzed using one-way ANOVA in GenStat (17th edition; VSN International, 2014) with block design included in the model. Tissue Ca and Mg concentration data were analyzed separately in each case, and tissue types were analyzed using separate ANOVA tests in the tissue partitioning experiment. For the preliminary phenotyping experiment, six replicate plants were analyzed for each genotype. For the tissue partitioning experiment, three samples, each comprising pooled samples from four replicate plants, were analyzed for each genotype. Means of different *A. thaliana* genotypes were compared using Least Significant Difference (LSD) functions in GenStat with differences considered significant at *P* < 0.05. Further LSD tests were conducted at *P* < 0.01 and *P* < 0.001 levels.

## Results

### Variation in Leaf Ca and Mg Concentration in the RIPR Diversity Population

The leaf concentrations of 21 mineral elements including Ca and Mg in the RIPR diversity population were previously determined by Thomas et al. (2016). Leaf Ca concentrations varied over threefold across the population, from 5,838 mg kg^-1^ to 18,752 mg kg^-1^. Leaf Mg concentrations were of a similar order of magnitude and varied over twofold, from 5,118 mg kg^-1^ to 13,429 mg kg^-1^. The frequency distribution of these two elements approximated a normal distribution (Supplementary Figure 6). Leaf Ca and Mg concentrations were among the highest, positively correlated elements measured across genotypes and tissues, with an *r*-value of 0.87 (*P* < 0.001). Leaf Ca and Mg concentrations varied between crop type, with higher concentrations of both elements in leaves of spring and semi-winter OSR than in winter OSR, winter fodder, and swede types.

### Associative Transcriptomics Suggest Flowering Time Regulators Are Important Markers for Leaf Ca and Mg Concentrations

**FIGURE 1:**
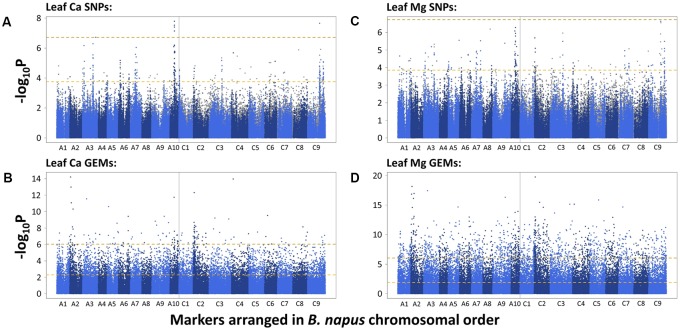
–log_10_P values of SNPs and GEMs associated with leaf Ca concentration (**A,B**, respectively) and leaf Mg concentration (**C,D**, respectively) in order of markers within the *B. napus* pan-transcriptome. Upper, gold, dashed line represents Bonferroni corrected significance threshold; lower, yellow, dashed line represents FDR corrected significance threshold (*P* = 0.05).

To identify candidate loci, SNPs and GEMs were used separately in analyses. A total of 1,295 and eight SNPs were found to be significantly associated with *B. napus* leaf Ca concentration after FDR and Bonferroni corrections, respectively. After removing SNPs with low second allele frequency, this was reduced to 247 and five SNPs, respectively, across all chromosomes. Visually determined association peaks on Manhattan plots were observed on chromosomes A3, A6, A7, A10, C2, C3, and C9 (**Figure 1A**). The most well defined peak was located on chromosome A10 and contained four out of the five SNPs above the Bonferroni corrected significance threshold (*P* = 0.05). The fifth SNP above this threshold fell in a peak on chromosome C9, in a region known to share sequence homology with parts of chromosome A10 (Chalhoub et al., 2014). A total of 5,557 and 141 GEMs were identified as significantly associated with leaf Ca concentration after FDR and Bonferroni corrections, respectively (**Figure 1B**). Notable peaks were observed on chromosomes A2 and C2. Single, associated GEMs were found at similar locations to SNP association peaks on chromosomes A3 and C2. The *A. thaliana* ortholog of *B. napus* genes corresponding to both these GEMs is At5g10140, which encodes FLOWERING LOCUS C (FLC), a transcription factor important for controlling flowering time (Michaels and Amasino, 1999). A further associated GEM was found in a region of chromosome A10, close to a SNP peak associated with leaf Ca concentration. Other single GEMs associated with leaf Ca concentration were observed on chromosomes A5, A6, C4, and C6. *B. napus* genes corresponding to these GEMs on chromosomes A5 and C4 are orthologous to *A. thaliana* At2g45660, which encodes SUPPRESSOR OF OVEREXPRESSION OF CO 1 (SOC1), another flowering time regulator (Lee et al., 2000).

The SNP and GEM associations for leaf Mg concentration were similar to those described for leaf Ca concentration. A total of 1,012 and one significant SNP(s) were found after FDR and Bonferroni corrections, respectively. After removing SNPs with low second allele frequency, this was reduced to 166 and zero SNPs, respectively, across all chromosomes. Of these 166 SNPs, 86 were identical to SNPs identified as significantly associated with leaf Ca concentration after FDR correction and removal of SNPs with low second allele frequency, indicating the potential of similar mechanisms to partly regulate accumulation. Visually determined association peaks largely co-localized with those associated with leaf Ca concentration, specifically on chromosomes A3, A7, A10, C2, C3, and C9 (**Figure 1C**). The most well defined peak was again on chromosome A10, and as before, a region on chromosome C9 with sequence homology to this region also contained associated SNPs. An association peak on chromosome A2 was also particularly well defined, containing 15 SNPs above the FDR corrected significance threshold. A total of 12,973 and 1,489 GEMs were identified as significantly associated with leaf Mg concentration after FDR and Bonferroni corrections, respectively, across all chromosomes (**Figure 1D**). Of these, 5,160 and 131 were also significantly associated with leaf Ca concentration after FDR and Bonferroni corrections, respectively. Notable peaks were again observed on chromosomes A2 and C2. The most highly associated GEMs on A3 and C2 were identical to those associated with leaf Ca concentration which correspond to *A. thaliana FLC*, and an associated GEM on C4 is identical to the GEM associated with leaf Ca concentration corresponding to *SOC1*.

### Genes Encoding Previously Identified Ca and Mg Transporters Are within Linkage Disequilibrium of Highly Associated Markers for Leaf Ca and Mg Concentration

Linkage disequilibrium describes the non-random association of alleles at different loci (Slatkin, 2008). Genes located physically near to each are generally inherited together, and hence are often in very strong LD. It is therefore feasible that any number of genes within LD of SNPs significantly associated with a trait may be controlling such associations. Based on associative transcriptomics results, seven loci were focussed on for the identification of candidate genes. These comprised regions of chromosomes A2, A3, A5, A6, A10, C2, and C4. A total of 17 *B. napus* candidate genes orthologous to 15 *A. thaliana* genes are summarized in **Table 2**. Four candidate genes were selected based on direct GEM hits as described above. These are Cab002472.4 and BnaC02g00490D (on chromosomes A3 and C2, respectively) encoding orthologs of *A. thaliana* At5g10140 (*FLC*), and Cab025356.1 and Bo4g024850.1 (on chromosomes A5 and C4, respectively) encoding orthologs of *A. thaliana* At2g45660 (*SOC1*). One and two candidate genes on chromosomes A2 and C2, respectively are orthologous to *A. thaliana MAGNESIUM TRANSPORTER 7* (*MGT7/MRS2-7*). This was previously characterized in *Arabidopsis thaliana* as an Mg transporter important for Mg uptake at low external concentrations (Gebert et al., 2009). A further candidate gene on chromosome A10 is orthologous to *A. thaliana AUTOINHIBITED CA^2+^-ATPASE, ISOFORM 8* (*ACA8*). This was previously characterized as a plasma membrane-localized Ca^2+^ transporting ATPase in *A. thaliana* (Bonza et al., 2000) and was identified as Ca responsive in *B. rapa* (Graham et al., 2014). The functions of the remaining nine candidate genes were selected based on sequence homology and annotations of *A. thaliana* orthologs and are either uncharacterized, or have not previously been experimentally shown to be involved in plant Ca or Mg accumulation (**Table 2**). These and At2g45660 (*SOC1*) were used for the selection of *A. thaliana* mutants.

**Table 2:** Summary of candidate genes selected from associative transcriptomics outputs.

Candidate	Chromosome	*A. thaliana*	Putative
gene		ortholog	function
Cab036107.1	A2	At5g09690	Mg transporter – MGT7
Cab039480.1	A2	At5g03960	IQ-domain – IQD12
Cab002472.4	A3	At5g10140	Flowering locus – FLC
Cab001235.1	A3	At2g05120	Nucleoporin – NUP133
Cab001274.1	A3	At2g13610	ABC transporter – ABCG5
Cab025356.1	A5	At2g45660	Suppressor of overexpression of CO – SOC1
Cab007043.1	A6	At5g48650	Nuclear transport factor – NTF2
Cab017470.1	A10	At5g57110	Ca transporting ATPase – ACA8
BnaC02g00490D	C2	At5g10140	Flowering locus – FLC
Bo2g007260.1	C2	At5g06530	ABC transporter – ABCG22
Bo2g008580.1	C2	At5g07320	ATP-Mg/Pi transporter – APC3
Bo2g009200.1	C2	At5g08670	Mitochondrial ATP synthase beta-subunit
Bo2g009480.1	C2	At5g09710	Mg transporter – MGT7
Bo2g009490.1	C2	At5g09690	Mg transporter – MGT7
Bo2g009910.1	C2	At5g10520	ROP binding protein kinase – RBK1
Bo2g011650.1	C2	At5g14040	Phosphate transporter – PHT3;1
Bo4g024850.1	C4	At2g45660	Suppressor of overexpression of CO – SOC1

*Putative functions obtained from The Arabidopsis Information Resource (TAIR; Huala et al., 2001)*.

### Four Mutant *A. thaliana* Lines Have Reduced Shoot Ca and Mg Concentration Compared to Wild Type and Effects Are Tissue Specific

**FIGURE 2:**
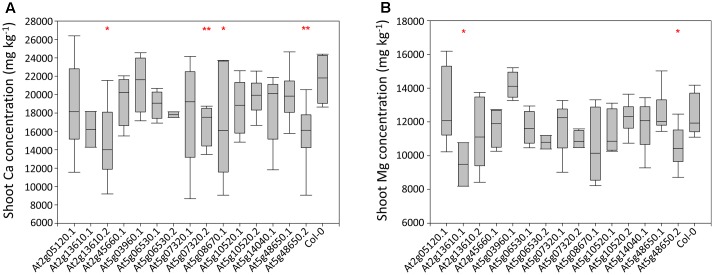
Shoot Ca **(A)** and Mg **(B)** concentrations across 15 mutant *A. thaliana* lines and wild type (Col-0) plants. Boxes represent the mid two quartiles with the median drawn; whiskers are the 95% confidence limits. Single and double stars above boxes represent significance at *P* < 0.05 and *P* < 0.01, respectively, compared to wild type (Col-0) plants.

Shoot Ca concentrations in a preliminary *A. thaliana* phenotyping experiment varied threefold between individual plants, from 8,684 to 26,387 mg kg^-1^ dry weight (DW; Supplementary Table 3). Much of this variation was observed within genotypes, with the largest variation observed in lines At5g7320.1 and At5g08670.1. Four mutant lines had significantly lower mean shoot Ca concentrations than wild type plants. These were At2g13610.2 (*P* < 0.05), At5g07320.2 (*P* < 0.01), At5g08670.1 (*P* < 0.05) and At5g48650.2 (*P* < 0.01; **Figure 2A**). Leaf Mg concentrations varied less, with twofold variation from 8,189 to 16,186 mg kg^-1^ DW observed between individual plants. Much of this variation was between genotypes. Two mutant lines had lower mean shoot Mg concentration than wild type plants. These were At2g13610.1 (*P* < 0.05) and At5g48650.2 (*P* < 0.05; **Figure 2B**). Based on these data, lines At2g13610.2, At5g07320.2, and At5g48650.2 were selected for characterization of tissue specific leaf Ca and Mg concentration.

**FIGURE 3:**
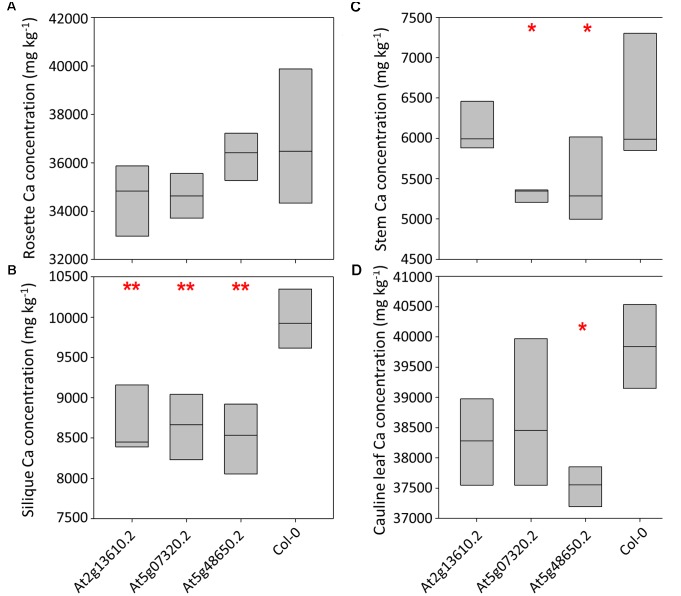
Rosette leaf **(A)**, silique **(B)**, stem **(C)** and cauline leaf **(D)** Ca concentrations across three mutant *A. thaliana* lines and wild type (Col-0) plants. Boxes represent full range of values with the median drawn. Single and double stars above boxes represent significance at *P* < 0.05 and *P* < 0.01, respectively, compared to wild type (Col-0) plants.

**FIGURE 4:**
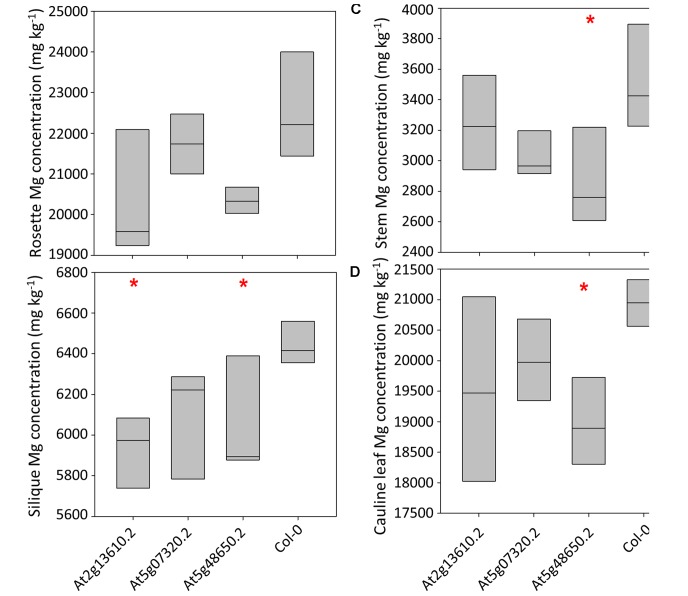
Rosette leaf **(A)**, silique **(B)**, stem **(C)** and cauline leaf **(D)** Mg concentrations across three mutant *A. thaliana* lines and wild type (Col-0) plants. Boxes represent full range of values with the median drawn. Single and double stars above boxes represent significance at *P* < 0.05 and *P* < 0.01, respectively, compared to wild type (Col-0) plants.

Calcium concentrations varied over eightfold between tissues and pooled tissue samples, ranging from 4,998 mg kg^-1^ DW in stems to 40,536 mg kg^-1^ DW in cauline leaves (**Figures 3A–D** and Supplementary Table 4). Cauline and rosette leaf Ca concentrations were similar, ranging from 32,966 mg kg^-1^ DW in rosette leaves to 40,536 mg kg^-1^ DW in cauline leaves. Mean silique Ca concentrations were lower in lines At2g13610.2, At5g07320.2, and At5g48650.2 than wild type plants (*P* < 0.01; **Figure 3B**). Mean stem Ca concentrations were lower in lines At5g07320.2 and At5g48650.2 than wild type plants (*P* < 0.05; **Figure 3C**). Mean cauline leaf Ca concentration was lower in line At5g48650.2 than wild type plants (*P* < 0.05; **Figure 3D**). Mg concentrations varied over ninefold between tissues and pooled samples, ranging from 2,608 mg kg^-1^ DW in stems to 23,999 mg kg^-1^ DW in rosette leaves (**Figures 4A–D**). Cauline leaf and rosette leaf Mg concentrations had a similar range, from 18,026 to 21,328 mg kg^-1^ DW and from 19,240 to 23,999 mg kg^-1^ DW, respectively. Lines At2g13610.2 and At5g48650.2 had lower mean silique Mg than wild type plants (*P* < 0.05; **Figure 4B**). Line At5g48650.2 also had lower mean stem Mg than wild type plants. Finally, and comparable with results from cauline leaf Ca concentration analysis, mean cauline leaf Mg concentration was lower in line At5g48650.2 than in wild type plants (*P* < 0.05; **Figure 4D**).

In summary, data from *A. thaliana* experiments identified four and two mutant lines with lower shoot Ca and Mg concentrations than wild type plants, respectively, and three of these mutations have tissue specific phenotypes. The main tissue specific effects were observed in silique tissue, with lower silique Ca concentrations in all three mutant lines investigated in the tissue partitioning experiment.

## Discussion

### SNP Based Association Analyses Identify Novel and Confirm Pre-determined Candidate Loci for Leaf Ca and Mg Concentrations

Leaf Ca concentration was highly associated with loci on chromosomes A3, A6, A7, A10, C2, C3, and C9 (**Figure 1A**). Similar loci were associated with leaf Mg concentration, specifically in regions of chromosomes A3, A7, A10, C2, C3, and C9 (**Figure 1C**). The most highly associated SNP for leaf Ca concentration was located on chromosome A10 (**Figure 1A**). This co-localizes with associated markers on C9 and markers on A10 and C9 for leaf Mg concentration. Co-localization of association peaks and associated markers for both mineral elements is unsurprising, as leaf Ca and Mg concentration data used in this study were very highly correlated (*r* = 0.87, *P* < 0.001; Thomas et al., 2016) and may reflect the relative lack of selectivity between these and other group II elements during accumulation within the plant (White, 2001). Such correlations between shoot Ca and Mg concentration have been previously shown in *B. oleracea* (Broadley et al., 2008) and a number of other angiosperm species (Broadley et al., 2004). Bus et al. (2014) previously investigated the genetic control of shoot ionome traits across 505 lines of *B. napus* using 3,910 SNPs in association analyses. Results showed two associations at a locus on chromosome C9 for shoot Ca and Mg concentration with a further association on chromosome C7 for shoot Ca concentration. The detection of an association locus on C9 is consistent with co-localized associations identified in this study. These results are also consistent with earlier findings by Broadley et al. (2008) who identified significant QTL for shoot Ca and Mg in *B. oleracea* on chromosomes C2, C6, C7, C8, and C9. Together, these results indicate the importance of loci on chromosomes A10 and C9 for Ca and Mg accumulation. The QTL identified for shoot Mg in *B. oleracea* on chromosome C2 by Broadley et al. (2008) is also consistent with findings in the present study that a locus on C2 is highly associated with leaf Mg concentration in *B. napus*. However, further work is required to confirm whether the loci are in close proximity to one another. To our knowledge, the remaining loci identified in this study have not been previously identified as important QTL for leaf Ca and Mg concentration in *Brassica* spp.

### FLC and SOC1 GEM Associations May Be Linked to Variation in Leaf Ca and Mg Concentrations between Spring and Winter *B. napus* Types in the RIPR Panel

Gene-expression marker analyses associated markers corresponding to *FLC* and *SOC1* with both leaf Ca and Mg concentrations. In *A. thaliana*, FLC is a repressor of flowering (Michaels and Amasino, 1999) and it has been previously shown that FLC transcript concentration correlates with vernalization requirements (Sheldon et al., 2000). Expression levels of *SOC1* also correlate with flowering time in *A. thaliana*; in lines which flower later, *SOC1* expression is very low (Lee et al., 2000). It is thought that *SOC1* expression is repressed by *FLC*, indicating the tight regulatory links between these genes and flowering time. Leaf Ca and Mg concentration data used in this study were obtained from analysis of plants in the RIPR panel (Thomas et al., 2016) which includes a large number of spring and winter *B. napus* varieties. Thomas et al. (2016) observed differences in leaf Ca and Mg concentrations between these types, with higher mean concentrations of both leaf Ca and Mg in spring OSR compared to winter OSR and winter fodder types. Since winter OSR types are generally considered to have longer vernalization requirements than spring types, it is possible that the association of *FLC* and *SOC1* with leaf Ca and Mg concentration observed in this study was a result of differences in vernalization requirement between these groups rather than direct genetic control of Ca and Mg uptake. It is worth noting that the association of GEMs with a trait does not indicate the causative polymorphism/s, only genes in which expression level is associated with variation in the trait. The causative polymorphism/s may lie in the promotor sequence of such genes, or localize somewhere upstream in the pathway. Hence, in the case of the flowering time genes identified here, it is unclear whether or not the observed associations with leaf Ca and Mg concentration are directly caused by changes in expression of *FLC* and *SOC1*. Despite this, their expression appears to be a suitable marker for the concentrations of these elements in *B. napus*. Further to this, the concentrations of a number of other mineral elements measured in the study of Thomas et al. (2016) were found to vary between crop types with typically different flowering times and vernalization requirements. Most notably, leaf concentrations of Mo, Na, P, and S were higher in spring OSR than winter OSR types. This suggests that flowering time, or the upstream mechanisms leading to changes in flowering time, has an effect on the concentrations of a number of nutrients in *B. napus*, though the pathway/s that lead to these differences remain unclear.

### ACA8 and MGT7 Are among Genes within Linkage Disequilibrium of Associated Loci

Identification of high-resolution loci influencing leaf Ca and Mg concentrations enabled locus-specific exploration of the *Brassica* pan-transcriptomes and other genome resources for candidate genes within LD of SNPs. LD is especially relevant to the efficacy of associative transcriptomics in the absence of a marker in a trait-controlling gene. LD decays relatively quickly in *B. napus* (Ecke et al., 2010; Harper et al., 2012), and this helps to reduce the number of possibilities when searching for candidate genes. However, in this study, typically hundreds of genes were still within previously estimated LD decay (around 1–2 cM on average; Ecke et al., 2010) of most candidate loci. Fortunately, well annotated browsers of *Brassica* A and C genomes are available at Ensembl Plants (Kersey et al., 2016), which enabled rapid identification of nearby genes in the reference sequences with links to functional annotation of *A. thaliana* orthologs.

Most notable genes identified using this workflow include an ortholog of *A. thaliana ACA8* near markers associated with leaf Ca on chromosome A10 and two orthologs of *A. thaliana MGT7* near markers associated with leaf Mg on chromosomes A2 and C2. *ACA8* encodes a Ca^2+^ transporting ATP-ase localized to the plasma membrane (Bonza et al., 2000). A *B. rapa* ortholog of *A. thaliana ACA8* was previously identified under an eQTL hot spot on chromosome A3 (Graham et al., 2014). The eQTL associated with this gene was defined as Ca-responsive, i.e., the direction of the eQTL changed under high Ca supply. The *A. thaliana* ortholog of *ACA8* was further investigated *in silico* in the same study using publically available phenotypic data at the PIIMs database (Baxter et al., 2007). This led to the identification of *ACA8* T-DNA knockout mutants with greater shoot Ca concentrations than control plants in over 50% of mutant samples, indicating the ability of this gene to influence Ca accumulation in *Brassica*. *MGT7* is a member of the MGT/MRS2 Mg transport family. This was previously characterized as a key transporter for Mg uptake at low external Mg concentrations by Gebert et al. (2009). *Arabidopsis thaliana* T-DNA knockout mutants were severely retarded in development when grown at low external Mg concentrations, but were visually unaffected when grown at higher external Mg concentrations. Both *ACA8* and *MGT7* are very promising candidate genes for the control of Ca and Mg accumulation in *B. napus*. The presence of these genes within LD of highly associated SNPs demonstrates the effectiveness of associative transcriptomics in candidate gene identification. Since *ACA8* and *MGT7* knockout mutants had previously been characterized in *A. thaliana*, they were not included in further experiments in this study.

### *Arabidopsis thaliana* Mutant Phenotyping Reveals New Candidates for Ca and Mg Accumulation

The preliminary *A. thaliana* phenotyping experiment identified four mutant lines with lower shoot Ca concentrations and two with lower shoot Mg concentrations than wild type plants. The most notable of these was At5g48650.2, the only line in which both shoot Ca and Mg was affected. The gene mutated in this line encodes NUCLEAR TRANSPORT FACTOR 2 (NTF2). This protein is proposed to function in the import of RAN, a multifunctional GTPase involved in nucleocytoplasmic transport (Zhao et al., 2006). It is the first time that it has been characterized with a shoot Ca and Mg phenotype in *A. thaliana*. Further investigation of this line showed that it had lower Ca and Mg concentration than wild type plants in all tissues except rosette leaves, suggesting it could be a promising candidate for manipulating the translocation of Ca and Mg to specific tissues in crop plants. At5g07320 encodes the ATP-Mg/Pi transporter APC3. Despite being annotated as an Mg/Pi transporter, mutants in this gene were only found to have reduced shoot Ca concentration. Tissue specific characterisation of this line showed silique and stem Ca concentrations were lower than wild type plants. This suggests that, at least in these conditions, the effects of the mutation are limited. However, effects at different external Ca or Mg concentrations might be different. A further line mutated in a gene encoding an ABC transporter was found to have both lower Ca and Mg concentrations in siliques compared to wild type plants. Identifying candidate genes controlling silique nutrient traits is particularly important in *B. napus*, which is mostly grown for the harvest of seeds which have a secondary use in animal feed. All *A. thaliana* experiments in this study took place using a high-nutrient compost. This could have masked the phenotypes of mutations in a number of candidate genes which may have been able to maintain normal Ca and Mg concentrations due to sufficient soil concentrations. In addition, it is possible that the mutations characterized here would show greater defects in shoot Ca and Mg concentrations when grown in nutrient limiting conditions. As well as this, all plants in both *A. thaliana* experiments were harvested at a single growth stage and other phenotypes might be seen at other growth stages. Despite this, four candidate genes analyzed here have proven to be potential targets for altering Ca and Mg concentrations in *B. napus*. These are orthologs of the *Arabidopsis thaliana* genes At2g13610, At5g07320, At5g08670, and At5g48650.

## Summary and Potential Applications

In this study, we have identified a number of genetic loci associated with leaf Ca and Mg concentration in *B. napus*. Within these loci, several novel candidate genes together with genes previously shown to influence or respond to Ca and Mg concentrations in this and closely related *Brassica* spp. were localized. Most well defined loci included regions on chromosomes A2, A10, C2, and C9, close to the known Ca and Mg transporters *ACA8* and *MGT7*. Experiments in *A. thaliana* T-DNA knockouts confirmed that a further four candidate genes influence shoot Ca and Mg concentrations. This study used *B. napus* associative transcriptomics followed by an *A. thaliana* T-DNA knockout workflow to identify and test candidate genes quickly and efficiently. Due to similar phylogeny, genes characterized here in *A. thaliana* are likely to have additive effects in *B. napus*. However, further study of candidate genes in *B. napus* is required to confirm *A. thaliana* gene functions observed here and in previous studies are conserved. Both *ACA8* and *MGT7* are good targets for this, especially since *ACA8* has previously exhibited Ca-responsiveness in *B. rapa* (Graham et al., 2014), and since the effects of mutations in *A. thaliana MGT7* are so marked. Selection of *B. napus* genotypes with different alleles of target genes may lead to improved ability to grow in the presence of low soil Ca or Mg concentrations. The development of high Ca and Mg accumulating lines in edible portions of *Brassica* spp. also has the potential to reduce nutrient deficiencies in humans and livestock across the world.

## Author Contributions

MB, IB, PW, TA, and NG conceived the project and contributed to experimental design. TA analyzed associative transcriptomics data and performed and analyzed *A. thaliana* mutant experiments. LH and ZH prepared functional genotypes and performed associative transcriptomics. TA and NG wrote the manuscript. All authors contributed to and have read and approved the final version of the manuscript.

## Conflict of Interest Statement

The authors declare that the research was conducted in the absence of any commercial or financial relationships that could be construed as a potential conflict of interest.

## Footnotes

1 https://bip.earlham.ac.uk/
