# Supplementary figures and images for: Identification of Candidate Genes for Calcium and Magnesium Accumulation in Brassica napus L. by Association Genetics
Source: Front Plant Sci. 2017 Nov 15;8:1968. doi: 10.3389/fpls.2017.01968 (PMC5694822; doi:10.3389/fpls.2017.01968)

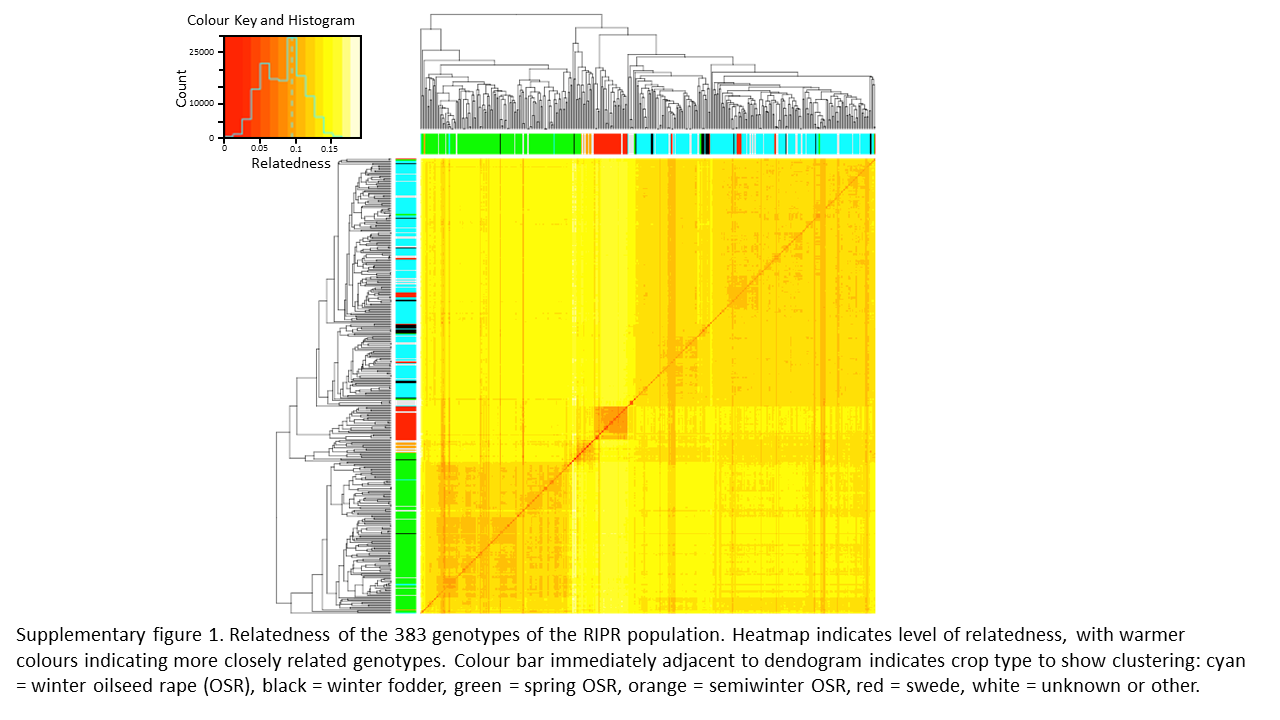

Supplement: Supplementary file 5 [file Image_1.TIF]

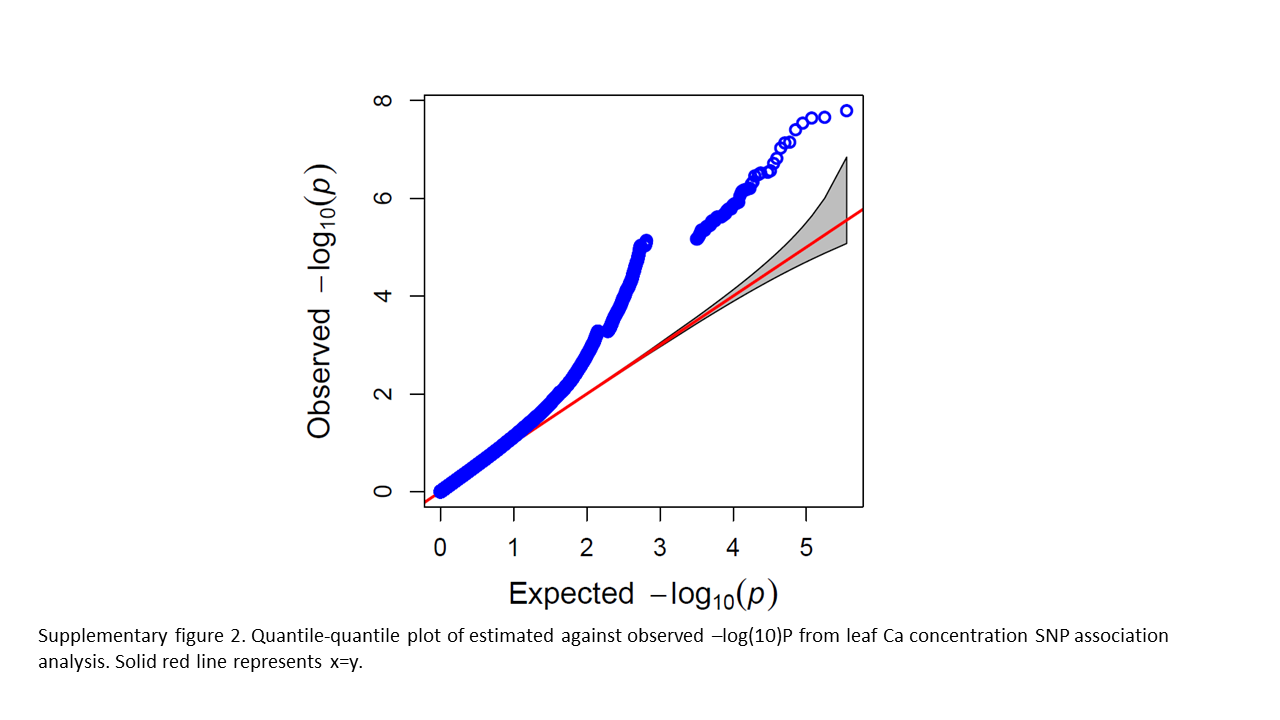

Supplement: Supplementary file 6 [file Image_2.TIF]

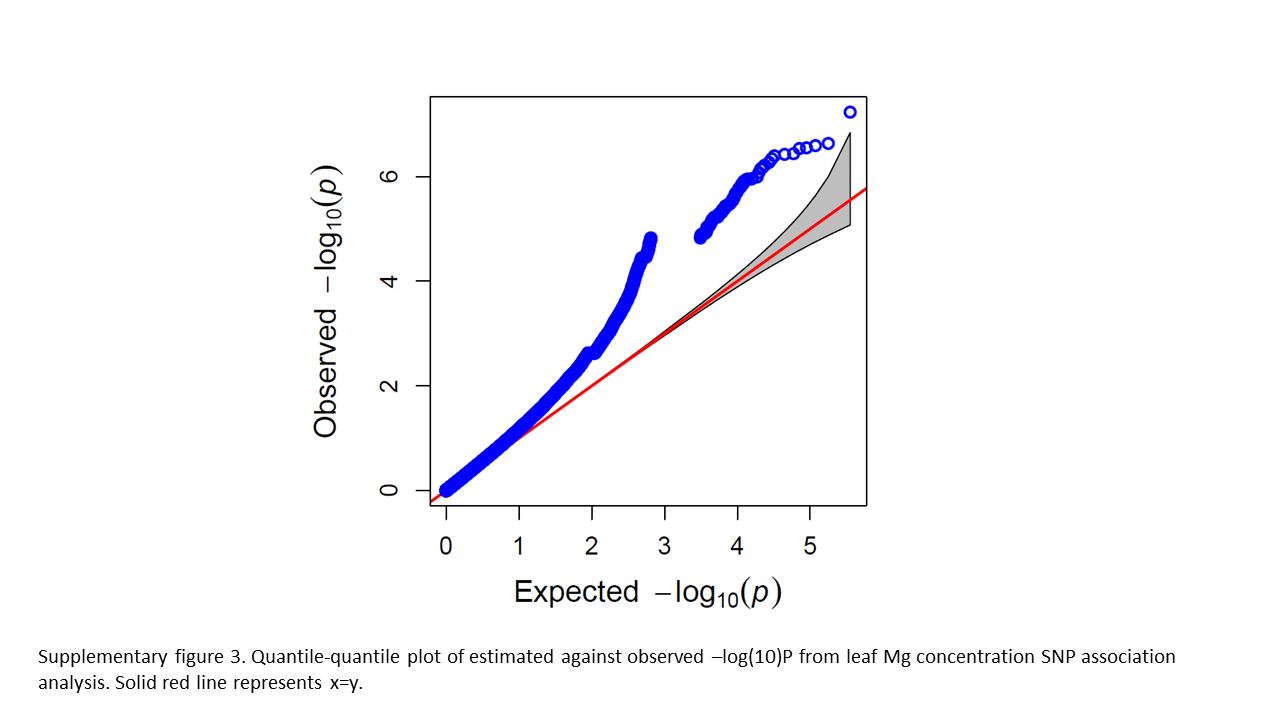

Supplement: Supplementary file 7 [file Image_3.TIF]

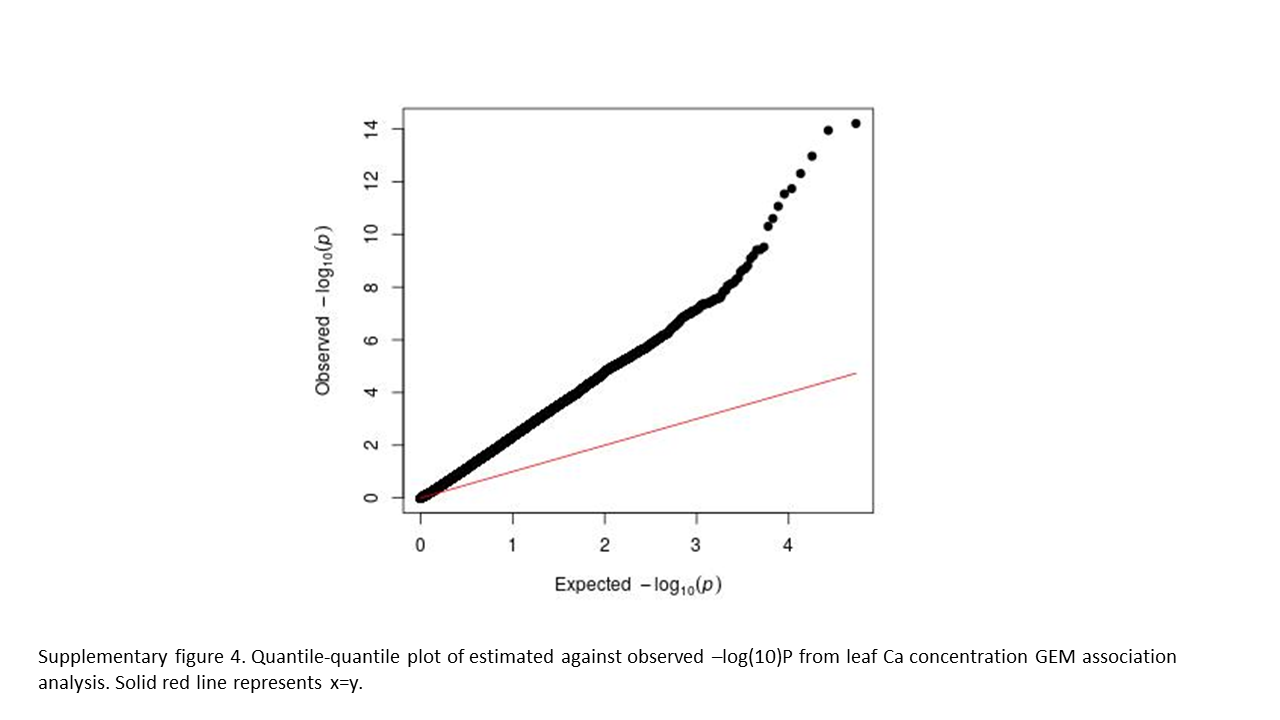

Supplement: Supplementary file 8 [file Image_4.TIF]

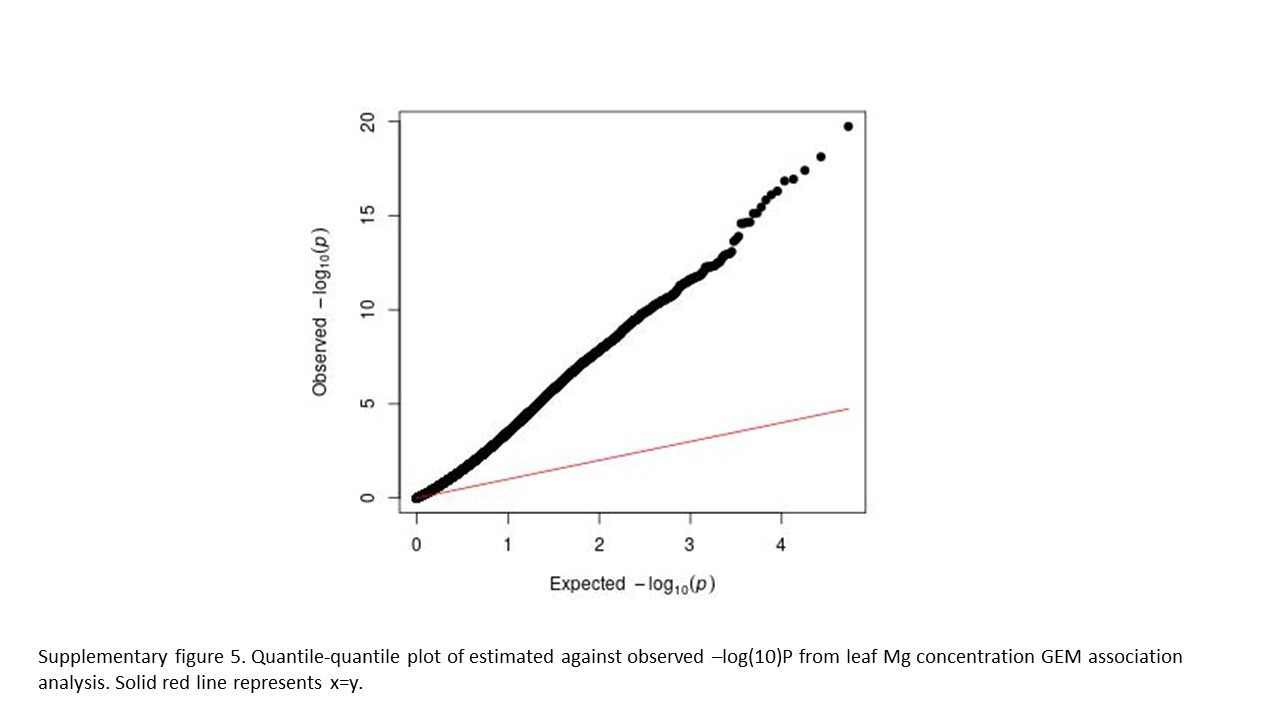

Supplement: Supplementary file 9 [file Image_5.TIF]

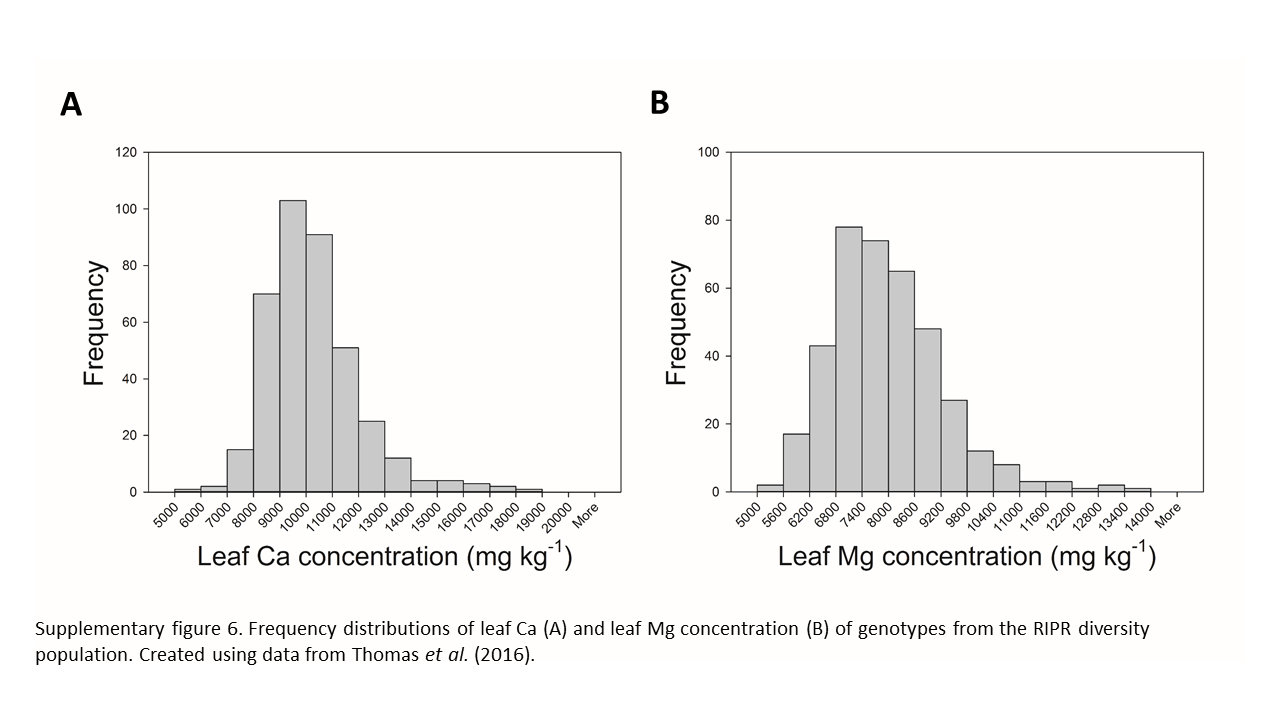

Supplement: Supplementary file 10 [file Image_6.TIF]
